# A Novel Gene Expression Scoring System Predicts Recurrence in Non‐Muscle‐Invasive Bladder Cancer Patients

**DOI:** 10.1002/cam4.70349

**Published:** 2024-11-14

**Authors:** Emina Kayama, Motohide Uemura, Akifumi Onagi, Satoru Meguro, Soichiro Ogawa, Kei Yaginuma, Kanako Matsuoka, Seiji Hoshi, Tomoyuki Koguchi, Junya Hata, Yuichi Sato, Hidenori Akaihata, Reiko Honma, Shinya Watanabe, Yoshiyuki Kojima

**Affiliations:** ^1^ Department of Urology Fukushima Medical University School of Medicine Fukushima Japan; ^2^ Department of Urology Iwase General Hospital Fukushima Japan; ^3^ Nippon Gene Co. Ltd. Tokyo Japan; ^4^ Translational Research Center Fukushima Medical University Fukushima Japan

**Keywords:** gene expression, microarray analysis, non‐muscle‐invasive bladder cancer, prognostic biomarker, recurrence risk

## Abstract

**Background:**

Despite the high recurrence rate of non‐muscle‐invasive bladder cancer (NMIBC), there are limitations in accurately predicting recurrence after transurethral resection of bladder tumor (TURBT) based on clinicopathological factors alone. However, prediction of recurrence using biomolecular characteristics of bladder tumors has not been applied to clinical practice. The objective of this study was to establish a new gene expression scoring system for identifying patients at high risk of recurrence.

**Methods:**

NMIBC and normal bladder samples were subjected to microarray analysis to obtain gene expression profiles. We identified 6 genes that were specifically upregulated in bladder cancer and also in recurrent cases. All patients were randomly grouped into a discovery cohort (*n* = 59) and a validation cohort (*n* = 30). Gene expression score (GES) was defined as the mean Z‐score of the 6 genes specific for recurrent bladder cancer.

**Results:**

The intravesical recurrence rate of the high GES group (*n* = 38) was higher than the low GES group (*n* = 21). GES was significantly associated with recurrence‐free survival in the validation cohort as well. In prognostic analysis, the European Organization for Research and Treatment of Cancer (EORTC) risk classification was not related to recurrence after TURBT in either univariate or multivariate analysis. On the other hand, the GES we developed was an independent factor for recurrence in NMIBC.

**Conclusions:**

A novel gene expression scoring system was shown to predict recurrence in NMIBC patients after TURBT and might be helpful in clinical decision‐making for NMIBC patients.

## Graphical abstract

We found six genes specific for recurrent bladder cancer and developed a new gene expression scoring system. Gene expression score was significantly associated with recurrence‐free survival in the validation cohort as well as the discovery cohort. This scoring system would be helpful in predicting recurrence and decision‐making for NMIBC patients.

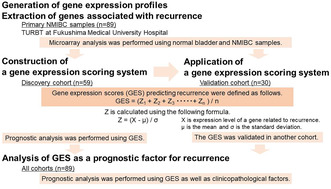

**Abbreviations**
ANLN: anillin
BCG: bacillus Calmette‐Guérin
CDCA3: cell division cycle‐associated protein 3
CI: confidence interval
CIS: concomitant carcinoma in situ
DHCR24: 24‐dehydrocholesterol reductase
EAU: European Association of Urology
EORTC: European Organization for Research and Treatment of Cancer
FC: fold change
GES: gene expression score
HR: hazard ratio
IQR: interquartile range
MIBC: muscle‐invasive bladder cancer
MTHFD2: methylenetetrahydrofolate dehydrogenase 2
NMIBC: non‐muscle‐invasive bladder cancer
RFS: recurrence‐free survival
STMN1: stathmin 1
TURBT: transurethral resection of bladder tumor
UC: urothelial carcinoma
XRCC2: X‐ray repair cross‐complementing 2.

## 1 Introduction

Bladder cancer is a common malignancy, with 573,278 new cases and 43,646 deaths reported in 2020 [1]. Approximately 75% of patients initially diagnosed with bladder cancer have non‐muscle‐invasive bladder cancer (NMIBC) [2]. Among NMIBC patients, 50%–75% relapse and 10%–15% progress to muscle‐invasive bladder cancer (MIBC) within 5 years [3, 4]. Bladder cancer is the most expensive cancer to treat because of the need for frequent and long‐term surveillance [5, 6].

Clinicopathological risk factors for disease recurrence and progression include tumor multiplicity, size, grade, stage, concomitant carcinoma in situ (CIS), and prior recurrence rate [3]. Risk factors are incorporated into the European Organization for Research and Treatment of Cancer (EORTC) risk classification, which is widely used in the clinical field [3, 7]. For patients with tumors presumed to be at low risk, one immediate chemotherapy vesical instillation after initial transurethral resection of bladder tumor (TURBT) is recommended. Patients with intermediate‐risk tumors should receive 1 year of full‐dose intravesical bacillus Calmette‐Guérin (BCG) immunotherapy or instillations of chemotherapy for a maximum of 1 year. For patients with high‐risk tumors, full‐dose intravesical BCG for 1–3 years is indicated [2]. Adjuvant intravesical instillations reduce the risk of recurrence [8] and delay or prevent progression to MIBC [9].

However, there are limitations in accurately predicting the prognosis of NMIBC based on clinical and pathological factors alone. Tumors with similar histopathological characteristics could have widely different molecular features and might belong to distinct molecular subgroups of varying aggressiveness. Although recent studies classified heterogenous NMIBC cases using biomolecular characteristics, such molecular subtypes have not yet been applied in clinical practice [10, 11, 12, 13]. Our aim here was to establish a new gene expression scoring system for identifying patients at high risk of recurrence.

## 2 Materials and Methods

### 2.1 Study Design

**FIGURE 1:**
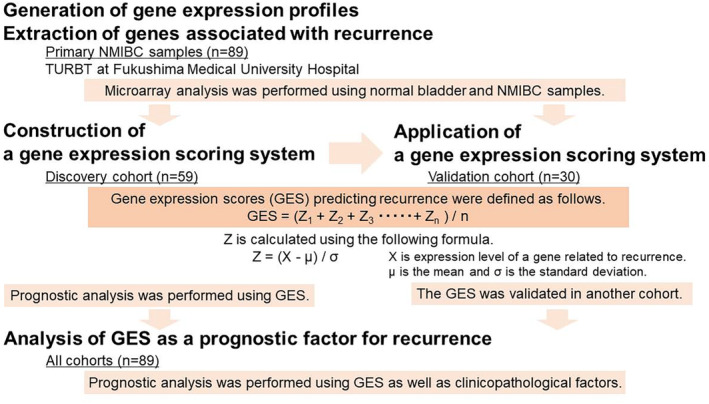
Study design. Eighty‐nine NMIBC samples and 8 normal bladder samples were subjected to microarray analysis. Gene expression profiles were obtained to extract genes associated with recurrence. All patients were randomly grouped into a discovery cohort and a validation cohort. GES were defined as the mean Z‐scores of genes specific for recurrent bladder cancer. Univariate and multivariate analysis in all patients was performed to prove that GES is a prognostic factor for recurrence.

The study design is shown in Figure 1. We included 89 NMIBC patients with no history of upper urinary tract urothelial carcinoma (UC) who underwent initial TURBT at Fukushima Medical University Hospital between May 2008 and January 2020. This study was approved by the ethics committee of Fukushima Medical University Hospital (#2097). All specimens were processed according to the standard pathological procedures and were histologically confirmed to be UC. Two or more experienced senior pathologists evaluated the pathological diagnosis according to the 2002 American Joint Committee on Cancer/Union Internationale Contre le Cancer TNM classification and graded according to the 2004 World Health Organization classification. Treatment strategies such as adjuvant intravesical instillations for NMIBC were based on the European Association of Urology (EAU) guidelines. Patients underwent cystoscopy and urinary cytology every 3 months for 2 years and then every 6 months thereafter. Computed tomography was performed annually for high‐risk NMIBC patients. The date of recurrence was defined as the date of pathologically proven recurrence by biopsy or TURBT.

### 2.2 Generation of Gene Expression Profiles

Eighty‐nine NMIBC samples and eight normal bladder samples were subjected to microarray analysis (to avoid mRNA amplification bias). A small fraction (3 × 3 mm) from each TURBT specimen was immediately frozen in liquid nitrogen. Total RNA was extracted from frozen tissue using ISOGEN (Nippon Gene, Tokyo, Japan); poly(A) + RNA was purified using MicroPoly(A)Purist kit (Ambion, Austin, TX, USA). RNA from patients was converted to cDNA using SuperScript II (Invitrogen, Carlsbad, CA, USA) and labeled with cyanine 5‐dUTP (PerkinElmer, Wellesley, MA, USA). Cyanine 3‐dUTP (PerkinElmer) was used to label cDNA converted from Human Universal Reference RNA Type I (MicroDiagnostic, Tokyo, Japan): poly(A) + RNA or Human Universal Reference RNA Type II (MicroDiagnostic): total RNA. Hybridization was performed with Labeling and Hybridization kit (MicroDiagnostic); signals were measured with GenePix 4000B Scanner (Axon Instruments, Union City, CA, USA) and GenePix Pro 3.0 software (Axon Instruments). The gene expression level value was log2 gene expression ratio normalized by GeneSpring GX v14.9 software package (Agilent Technologies, Santa Clara, CA, USA).

### 2.3 Construction of a Gene Expression Scoring System

All patients were randomly grouped into a discovery cohort (*n* = 59) and a validation cohort (*n* = 30). In the discovery cohort, we constructed a gene expression scoring system predicting the recurrence of NMIBC after TURBT. A gene expression score (GES) was defined as the mean Z‐scores of genes specific for recurrent bladder cancer, with Z‐score representing the number of standard deviations a gene's expression level is away from the mean of samples. The gene expression scoring system derived in the discovery cohort was applied to the validation cohort to confirm its reproducibility.

### 2.4 Statistical Analysis

Categorical and continuous variables were compared between the discovery and validation cohort using chi‐square test and the Mann–Whitney *U* test, respectively. In each cohort, recurrence‐free survival (RFS) curves were generated by the Kaplan–Meier method, starting from the date of TURBT. The difference in RFS rate between the two groups (high versus low GES) was compared using the log‐rank test and considered statistically significant when the *p* < 0.05. To assess the relative contributions of GES and clinicopathological factors for RFS, univariate and multivariate analysis in all patients was performed by the Cox proportional hazards model. The independent variables included in multivariate analysis were EORTC risk classification (high + intermediate vs. low risk) and GES (high vs. low). All statistical analyses were performed by SPSS v27.0 software (IBM SPSS, Chicago, IL, USA).

### 2.5 Analysis Using Public Database

Expression levels of 6 selected genes in normal and bladder cancer tissues were analyzed using the web tool TNMplot.com (https://tnmplot.com/analysis/; accessed on 10 January 2024). This web tool contained data generated by RNA‐seq from The Cancer Genome Atlas, Therapeutically Applicable Research to Generate Effective Treatments, and The Genotype‐Tissue Expression repositories [14]. We compared RNA expression values between normal (*n* = 19) and bladder urothelial carcinoma samples (*n* = 19).

### 2.6 Immunohistochemical Analysis

The expression of the 6 genes was assessed through immunohistochemical staining of paraffin‐embedded tissues from NMIBC or normal bladder specimens. Formalin‐fixed paraffin‐embedded sections (5‐μm thick) were deparaffinized and rehydrated. Endogenous peroxidase activity was blocked using 0.3% H_2_O_2_ followed by boiling the slides for 10 min in citrate buffer (pH 6.0) or Tris‐ethylenediaminetetraacetic acid (EDTA) (pH 9.0) for antigen retrieval. Nonspecific binding of immunoglobulin G was blocked using 5% skim milk. Primary antibodies against cell division cycle‐associated protein 3 (CDCA3; 1:50; 15,594‐1‐AP; Proteintech, Rosemont, USA), X‐ray repair cross‐complementing 2 (XRCC2; 1:50; sc‐365,854; Santa Cruz Biotechnology, Dallas, TX, USA), anillin (ANLN; 1:50; HPA005680; Atlas Antibodies, Bromma, Sweden), methylenetetrahydrofolate dehydrogenase 2 (MTHFD2; 1:250; ab151447; Abcam, Cambridge, UK), stathmin 1 (STMN1; 1:500; ab52630; Abcam, Cambridge, UK), and 24‐dehydrocholesterol reductase (DHCR24; 1:200; HPA063005; Atlas Antibodies, Bromma, Sweden) were applied and incubated overnight at 4°C. The N‐Histofine Simple Stain MAX PO (Nichirei Biosciences, Tokyo, Japan) was utilized for the peroxidase‐labeled secondary antibody reaction. Sections were then stained with 3,3′‐diaminobenzidine tetrahydrochloride and counterstained with hematoxylin. Subsequently, they were dehydrated through a graded ethanol series, cleared in xylene, and mounted with coverslips.

## 3 Results

### 3.1 Clinicopathological Characteristics of NMIBC Patients

The median age of patients was 75.8 years (interquartile range [IQR]: 66.1–82.3 years). Men accounted for 79.8% of patients (*n* = 71) and women for 20.2% (*n* = 18). Table 1 shows the clinicopathological characteristics of the discovery cohort (*n* = 59) and validation cohort (*n* = 30). No significant differences were found in the rate of adjuvant intravesical therapy, observation period, and recurrence rate, nor in the pathology of TURBT specimens. BCG treatment was given to 23.7% of the discovery cohort (*n* = 14) and 20.0% of the validation cohort (*n* = 6) (*p* = 0.690); intravesical chemotherapy was provided in 30.5% (*n* = 18) and 46.7% (*n* = 14) of cases, respectively (*p* = 0.133). The median follow‐up periods after TURBT were 18.9 months (IQR: 3.5 to 43.9 months) in the discovery cohort and 15.6 months (IQR: 6.7 to 37.8 months) in the validation cohort (*p* = 0.938). Sixteen patients (27.1%) in the discovery cohort showed intravesical recurrence, while 7 (23.3%) in the validation cohort relapsed (*p* = 0.700).

**TABLE 1:** Clinicopathological characteristics of primary NMIBC patients.

Characteristics	Total		Discovery cohort		Validation cohort		*p*
	(*n* = 89)		(*n* = 59)		(*n* = 30)		
Age, median (IQR), years	75.8	(66.1–82.3)	75.5	(64.7–82.9)	76.2	(67.7–82.3)	0.931
Gender, *n* (%)							0.248
Male	71	(79.8%)	45	(76.3%)	26	(86.7%)	
Female	18	(20.2%)	14	(23.7%)	4	(13.3%)	
Urine cytology, *n* (%)							0.816
Class I, II, III	49	(55.1%)	33	(55.9%)	16	(53.3%)	
Class IV, V	40	(44.9%)	26	(44.1%)	14	(46.7%)	
Multiplicity, *n* (%)							0.199
Single tumor	36	(40.4%)	26	(44.1%)	9	(30.0%)	
Multiple tumors	54	(60.7%)	33	(55.9%)	21	(70.0%)	
Size, *n* (%)							0.690
≥ 3 cm	20	(22.5%)	14	(23.7%)	6	(20.0%)	
< 3 cm	69	(77.5%)	45	(76.3%)	24	(80.0%)	
Grade, *n* (%)							0.931
High grade	51	(57.3%)	34	(57.6%)	17	(56.7%)	
Low grade	38	(42.7%)	25	(42.4%)	13	(43.3%)	
Stage, *n* (%)							0.706
pTa	56	(62.9%)	36	(61.0%)	20	(66.7%)	
pT1	32	(36.0%)	22	(37.3%)	10	(33.3%)	
pTis	1	(1.1%)	1	(1.7%)	0	(0.0%)	
Concomitant CIS, *n* (%)							0.336
Yes	6	(6.7%)	5	(8.5%)	1	(3.3%)	
No	83	(93.3%)	54	(91.5%)	29	(96.7%)	
Squamous differentiation, *n* (%)							0.450
Yes	5	(5.6%)	4	(6.8%)	1	(3.3%)	
No	84	(94.4%)	55	(93.2%)	29	(96.7%)	
EORTC risk, *n* (%)							0.207
Low	14	(15.7%)	9	(15.3%)	5	(16.7%)	
Intermediate	23	(25.8%)	12	(20.3%)	11	(36.7%)	
High	52	(58.4%)	38	(64.4%)	14	(46.7%)	
Adjuvant BCG treatment							0.690
Yes	20	(22.5%)	14	(23.7%)	6	(20.0%)	
No	69	(77.5%)	45	(76.3%)	24	(80.0%)	
Adjuvant intravesical chemotherapy							0.133
Yes	32	(36.0%)	18	(30.5%)	14	(46.7%)	
No	57	(64.0%)	41	(69.5%)	16	(53.3%)	
Observation period, median (IQR), months	16.8	(3.8–42.6)	18.9	(3.5–43.9)	15.6	(6.7–37.8)	0.938
Recurrence							0.700
Yes	23	(25.8%)	16	(27.1%)	7	(23.3%)	
No	66	(74.2%)	43	(72.9%)	23	(76.7%)	

Abbreviations: BCG, bacillus Calmette‐Guérin; CIS, carcinoma in situ; EORTC, European Organization for Research and Treatment of Cancer; IQR, interquartile range; NMIBC, non‐muscle invasive bladder cancer.

### 3.2 Identification of Recurrent Bladder Cancer‐Specific Genes

**FIGURE 2:**
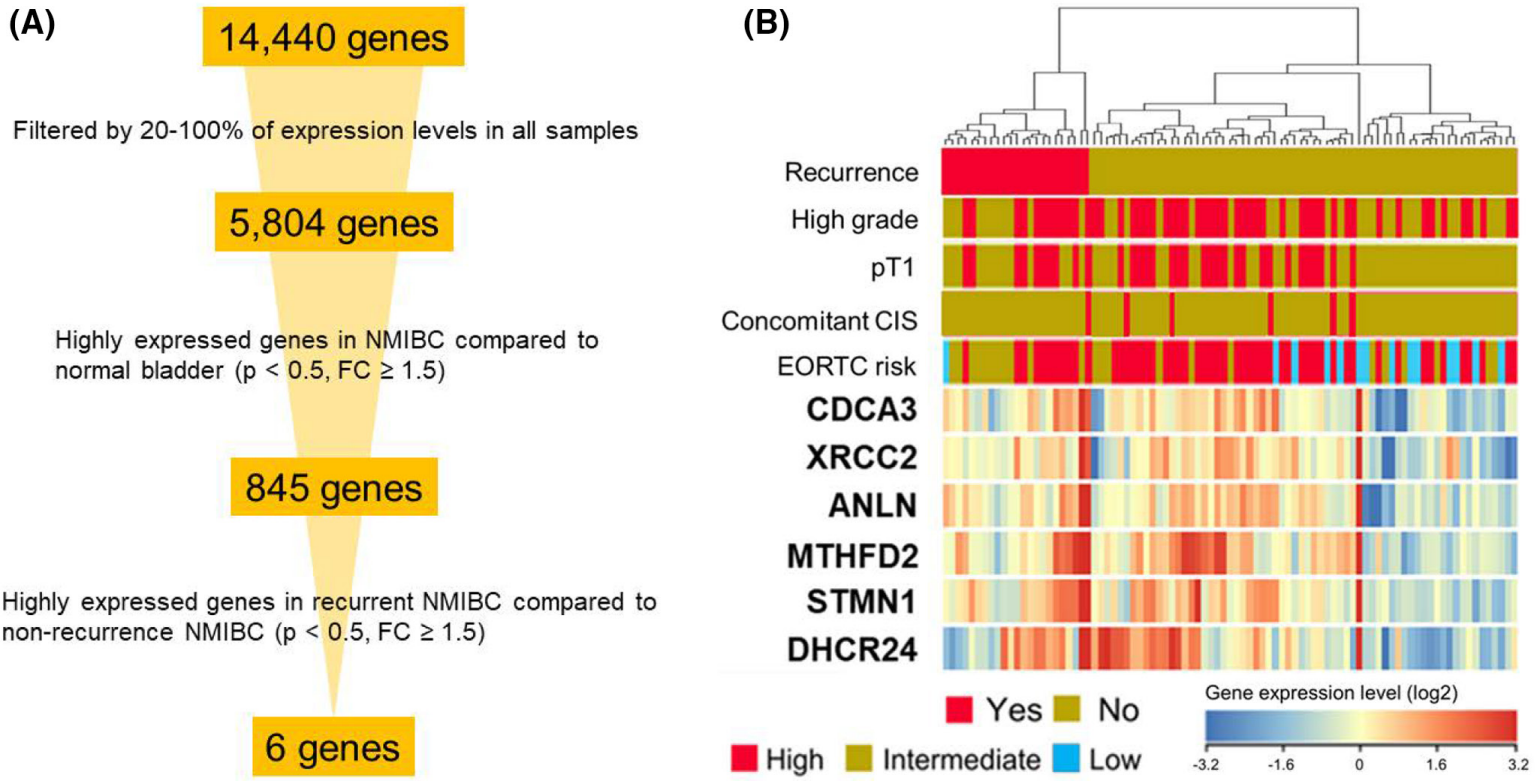
Identification of recurrent bladder cancer‐specific genes. (A) The algorithm for extracting the 6 genes that were highly expressed in bladder cancer and also especially upregulated in recurrent cases. (B) Expression patterns of the 6 genes in 89 tumor samples. Six genes consisted of CDCA3, XRCC2, ANLN, MTHFD2, STMN1, and DHCR24.

We performed a microarray analysis of tumor tissue from 8 non‐cancer and 89 primary NMIBC patients followed by supervised hierarchical cluster analysis. Figure 2A depicts the algorithm for extracting genes that were specifically expressed in NMIBC and associated with recurrence. From the initial 14,440 genes, genes with expression levels below 20% were removed, leaving 5804 genes. By volcano plots, 845 genes were overexpressed in NMIBC compared to normal bladder (*p* < 0.5, fold change [FC] ≥ 1.5). Finally, 6 genes that were expressed more highly in recurrent NMIBC than in non‐recurrent NMIBC were selected (*p* < 0.5, FC ≥ 1.5). FCs for each of the 6 genes and hazard ratios obtained from univariate Cox regression analyses of disease recurrence in 89 patients are displayed in Table S1. A hierarchical cluster analysis by supervised method in 89 tumor samples is shown in Figure 2B. The expression of the 6 selected genes tended to be higher in recurrent bladder cancer.

### 3.3 Disease Recurrence in the Discovery and Validation Cohort

**FIGURE 3:**
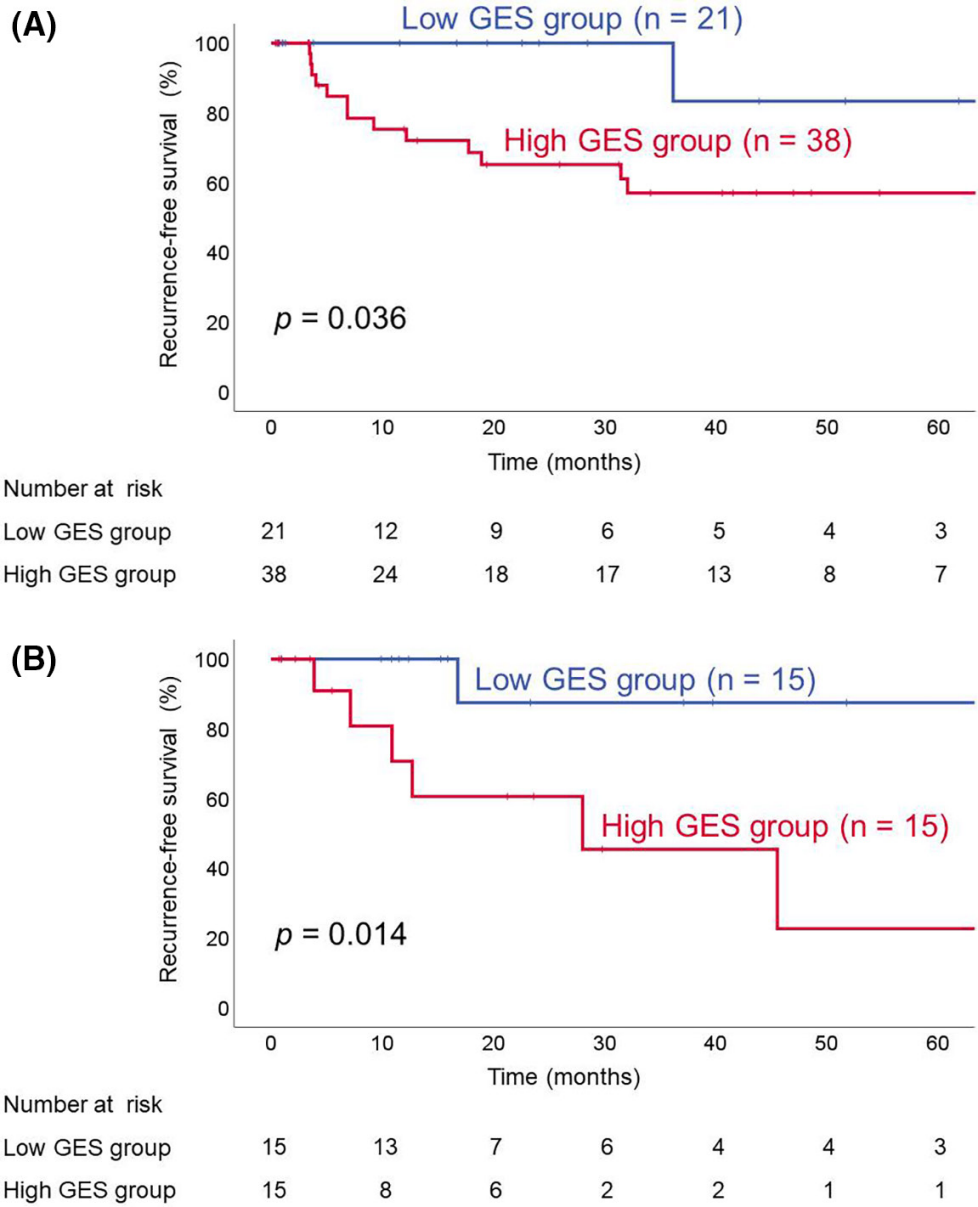
GES and recurrence‐free survival using Kaplan–Meier survival estimates. (A) Discovery cohort (*n* = 59). (B) Validation cohort (*n* = 30).

The discovery cohort was divided into high (*n* = 38) and low (*n* = 21) GES groups. A Kaplan–Meier curve demonstrated that the 2‐year RFS rate was 65.2% in the high GES group, significantly lower than for the low GES group (100%, *p* = 0.036, Figure 3A). We also compared RFS between the high (*n* = 15) and low (*n* = 15) GES groups in the validation cohort. As shown in Figure 3B, the high GES group had worse 2‐year RFS (60.6%) than the low GES group (87.5%, *p* = 0.014). Thus the GES constructed in the discovery cohort correlated with disease recurrence in the validation cohort as well.

### 3.4 Prognostic Factors in all Cohorts

In all cohorts, including a discovery cohort and a validation cohort, prognostic analysis was performed using clinicopathological factors and GES (Table 2). The univariate Cox's regression analysis indicated that high GES was related to disease recurrence (*p* = 0.006), while clinical characteristics and pathology were not. EORTC risk classification (high + intermediate vs. low risk), representing clinicopathological features, was not associated with recurrence after TURBT in either univariate (*p* = 0.127) or multivariate analysis (hazard ratio [HR] = 2.58, 95% confidence interval [CI] = 0.34–19.70, *p* = 0.362). On the other hand, the GES we developed was an independent factor for the recurrence of NMIBC in the multivariate analysis as well (HR = 6.50, 95% CI = 1.49–28.31, *p* = 0.013).

**TABLE 2:** Univariate and multivariate Cox regression analyses of disease recurrence in 89 patients.

Characteristics	Univariate analysis			Multivariate analysis		
	HR	95% CI	*p*	HR	95% CI	*p*
Age (≥ 75 years vs. < 75 years)	0.75	0.33–1.73	0.503			
Sex (male vs female)	1.39	0.51–3.85	0.515			
Urine cytology (class IV, V vs class I, II, III)	2.30	0.94–5.63	0.068			
Multiplicity (multiple tumors vs. single tumor)	2.16	0.80–5.83	0.128			
Size (≥ 3 cm vs. < 3 cm)	0.81	0.30–2.17	0.669			
Grade (high vs. low)	0.80	0.35–1.82	0.589			
Stage (pT1 + pTis vs. pTa)	1.07	0.47–2.46	0.866			
Concomitant CIS (yes vs. no)	0.27	0.03–2.09	0.209			
EORTC risk (high vs. intermediate + low)	0.83	0.55–1.25	0.367			
EORTC risk (high + intermediate vs. low)	4.78	0.64–35.51	0.127	2.58	0.34–19.70	0.362
Adjuvant BCG treatment (no vs. yes)	1.30	0.51–3.33	0.579			
Adjuvant intravesical chemotherapy (no vs. yes)	0.53	0.22–1.24	0.143			
GES (high vs. low)	7.64	1.79–32.64	**0.006**	6.50	1.49–28.31	**0.013**

*Note:* The bold values signify the *p* < 0.05. Abbreviations: BCG, bacillus Calmette‐Guérin; CI, confidence interval; CIS, carcinoma in situ; EORTC, European Organization for Research and Treatment of Cancer; GES, gene expression scores; HR, hazard ratio.

### 3.5 Transcriptome‐Level Expression of the 6 GES Genes in Public Databases

The results of the analysis using TNMplot.com are shown in Figure S1. In public databases obtained from RNA‐Seq, expression levels of the 6 genes were significantly higher in bladder cancer tissue than in adjacent normal tissue.

### 3.6 Protein Expression of 6 Genes in TURBT Specimens

**FIGURE 4:**
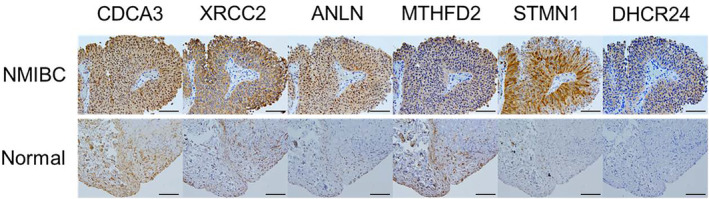
Immunohistochemical analysis of 6 genes in paraffin‐embedded tissues from NMIBC or normal bladder: CDCA3, XRCC2, ANLN, MTHFD2, STMN1, and DHCR24. Black scale bars, 50 μm.

Immunohistochemical staining was performed on NMIBC and normal bladder tissue to determine whether the increased mRNA expression of the six GES genes correlated with increased protein levels as well (Figure 4). The results indeed indicated elevated protein levels of the six genes, corroborating the RNA results shown by both microarray and RNA‐seq.

## 4 Discussion

We identified a specific subset of genes that predicted recurrence in NMIBC patients after TURBT and developed a novel gene expression scoring system. Because NMIBC comprises a heterogeneous group of patients with an enormous array of clinical and pathological risk factors involved, any effective multivariate analysis must include these factors to avoid confounding effects. Indeed, in our study, EORTC risk classification was not independently associated with recurrence after TURBT. EORTC risk classification, though commonly used in clinical practice, was based on seven clinical trials [3] more than 15 years ago. The clinical trials included patients who had already undergone intravesical therapy, and the treatment of NMIBC has changed over the years. Recently, the EAU NMIBC 2021 scoring model was constructed and the EAU guideline risk classification was updated [15], but these models intend to predict the risk of NMIBC progression, not recurrence. Our gene expression scoring system was based on gene expression profiles and may provide a more nuanced view of an individual cancer's characteristics. In addition, it was validated in another cohort, therefore, we believe it is highly reproducible.

Recent advances in microarray technology and next‐generation sequencing have led to progress in identifying molecular markers to predict progression and disease outcome in NMIBC [16, 17]. With regard to microarray studies, Dyrskjøt et al. reported an 88‐gene signature in NMIBC samples from 294 patients that predicted progression to MIBC independently from standard clinical risk variables [18]. The same authors showed a 12‐gene signature based on polymerase chain reaction significantly predicted disease progression in NMIBC [19], followed by a prospective multicenter validation study using a 12‐gene progression score [20]. However, progression from NMIBC to MIBC is rare, with Dyrskjøt et al. stating that progression rate was 5% [20]. Earlier studies had reported slightly higher progression rates, but patients were recruited during the 1980s, when treatment and follow‐up differed from current practice [3]. The two‐pathway model of UC classifies tumors into two categories, based on their modes of carcinogenesis, each with distinct histopathological and molecular features [4]. One category comprises non‐invasive tumors which recur frequently but are genetically stable, and the other is invasive tumors which are genetically unstable and accumulate many genomic alterations. Although recent studies [10, 11, 12, 13] have identified multiple molecular subtypes across the two‐pathway model, the heterogeneity of bladder cancer is difficult to fully elucidate. Whatever the subtype of NMIBC, the recurrence rate of bladder cancer is high, and predicting recurrence from TURBT specimens remains a clinical challenge.

We identified the following 6 genes as predictors of recurrence in patients with NMIBC: CDCA3, XRCC2, ANLN, MTHFD2, STMN1, and DHCR24. With the exception of XRCC2, 5 of these 6 genes have previously been reported to be associated with bladder cancer prognosis [21, 22, 23, 24, 25, 26, 27, 28, 29, 30, 31, 32, 33, 34]. CDCA3, ANLN, STMN1, and DHCR24 play important roles in cell proliferation and migration of bladder cancer. Expression of MTHFD2 in bladder cancer tissues correlates with PD‐L1 expression, which was proposed to be mediated by the PI3/AKT and JAK/STAT pathways. XRCC2 is a homologous recombination‐related gene implicated in a variety of cancers [35, 36, 37, 38]. Xu et al. showed XRCC2 overexpression inhibited colorectal cancer cell apoptosis and promoted proliferation by enriching cells in the G0/G1 phase [35]. Although the identification of genes linked to disease recurrence suggests potential therapeutic interventions based on their mechanisms of action, the lack of a biological context for these genes does not diminish their potential as clinical biomarkers. In our study, GES was defined as the mean Z‐scores of 6 gene expression level values, as using the Z‐scores enabled representing the different genes equally in the score despite the genes differing in mean levels of expression [39]. Since the GES obtained from microarray analysis is easy and cost‐effective, we could use GES as a biomarker in the clinical field. Furthermore, immunohistochemical analysis of pathological specimens confirmed the overexpression of the 6 genes on the protein level, potentially paving the way for more convenient personalized medicine following TURBT.

Patients diagnosed with NMIBC have a high recurrence rate, often with multifocal tumor occurrence, and tumors are thought to develop via field cancerization of the bladder [40]. This is supported by the observation that recurrent tumors share multiple clonal mutations [41, 42, 43]. Although a few patients develop multiple tumors that apparently evolved independently, tumors from the same patient are commonly related. Furthermore, tumors with the highest genomic complexity are not necessarily the last to appear [41, 44, 45], which provides an explanation for the observation that recurrent tumors may be lower grade than preceding tumors [46]. Even though only primary tumors were included in the current study, we expect that multiple testing points during the disease course will provide more robust estimates of disease recurrence over time.

There are several limitations in the present study. Due to a retrospective investigation, the selection of regimen and period of administration of adjuvant intravesical therapy were not unified. However, no significant differences were found in the rates of adjuvant BCG treatment and adjuvant intravesical chemotherapy between the discovery cohort and the validation cohort. Another limitation was that we used a random split method with a small cohort. Prospective validation with a different cohort may be necessary in the future. In addition, immunohistochemical staining was performed in only a small number of cases in this study. To facilitate the realization of personalized medicine, immunohistochemical staining needs to be quantified and proven to be associated with bladder cancer prognosis.

In summary we developed a novel gene expression scoring system to predict recurrence in NMIBC patients after TURBT. GES could be applied to improve clinical decision‐making; shortening the cystoscopy interval and BCG maintenance therapy might be beneficial for NMIBC patients with high GES.

## Author Contributions

**Emina Kayama:** data curation (equal), formal analysis (equal), writing – original draft (equal). **Motohide Uemura:** project administration (equal), writing – review and editing (equal). **Akifumi Onagi:** methodology (equal). **Satoru Meguro:** methodology (equal). **Soichiro Ogawa:** methodology (equal). **Kei Yaginuma:** investigation (equal). **Kanako Matsuoka:** investigation (equal). **Seiji Hoshi:** investigation (equal). **Tomoyuki Koguchi:** investigation (equal). **Junya Hata:** investigation (equal). **Yuichi Sato:** investigation (equal). **Hidenori Akaihata:** investigation (equal). **Reiko Honma:** investigation (equal). **Shinya Watanabe:** investigation (equal). **Yoshiyuki Kojima:** supervision (equal).

## Ethics Statement

This study was conducted in accordance with the principles of the Declaration of Helsinki. The ethics committee of Fukushima Medical University Hospital approved our study and written informed consent was obtained from all patients prior to enrolment (#2097).

## Consent

The authors have nothing to report.

## Conflicts of Interest

The authors declare no conflicts of interest.

## Supporting information


Figure S1.

Table S1.

**Data S1**.

## Data Availability

The datasets generated and/or analyzed during this study are available from the corresponding author upon reasonable request.
