# Supplementary figures and images for: A Novel Gene Expression Scoring System Predicts Recurrence in Non‐Muscle‐Invasive Bladder Cancer Patients
Source: Cancer Med. 2024 Nov 14;13(22):e70349. doi: 10.1002/cam4.70349 (PMC11561421; doi:10.1002/cam4.70349)

(A)

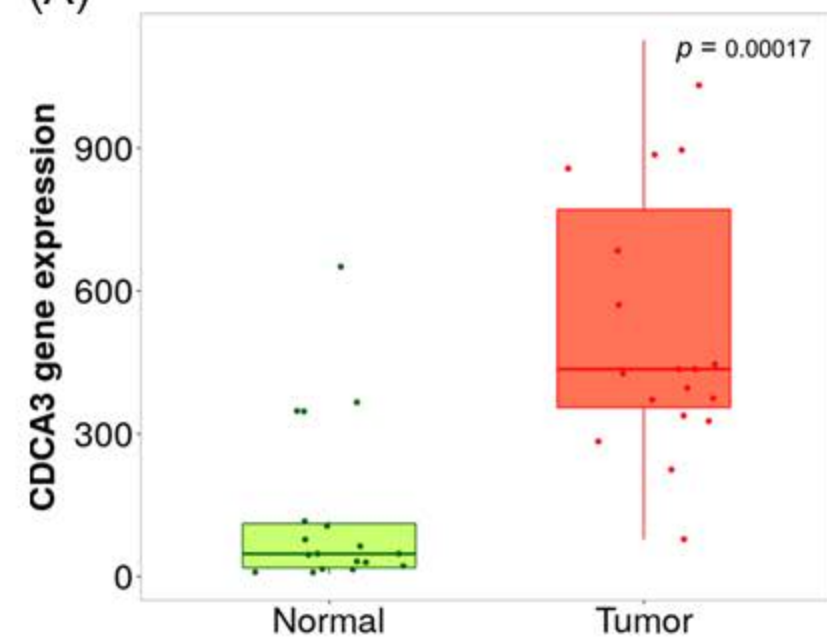

(B)

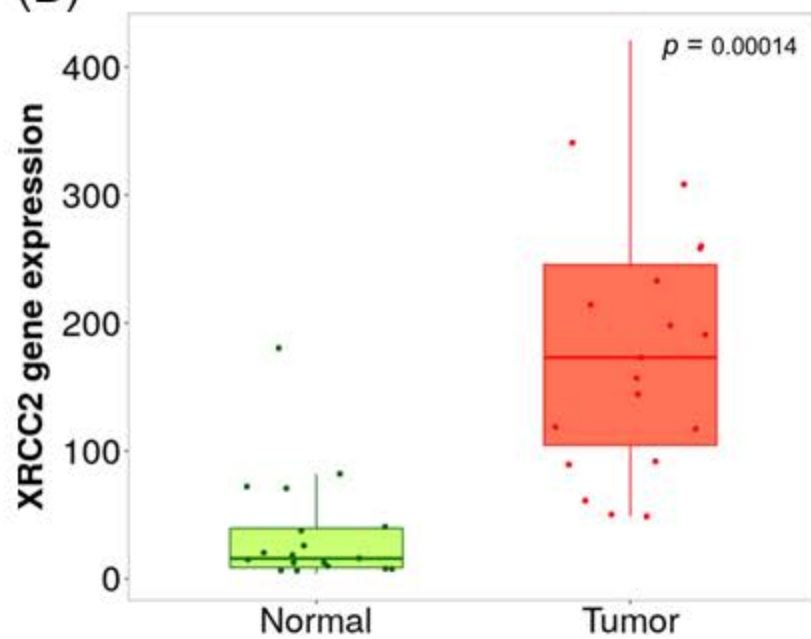

(C)

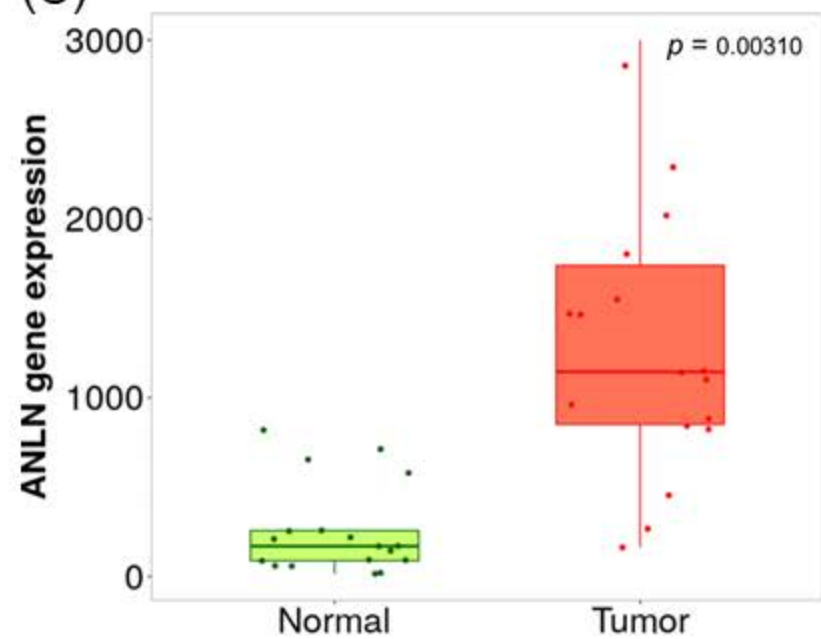

(D)

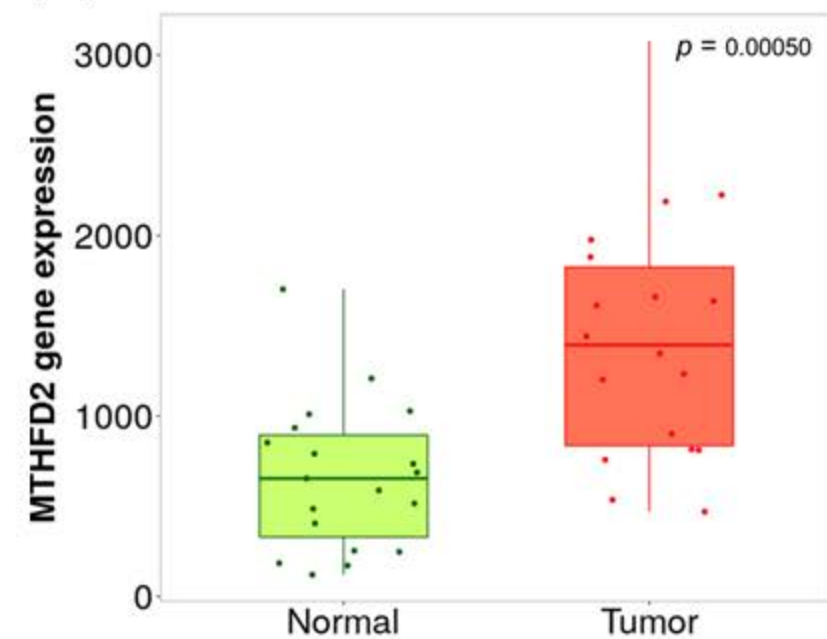

(E)

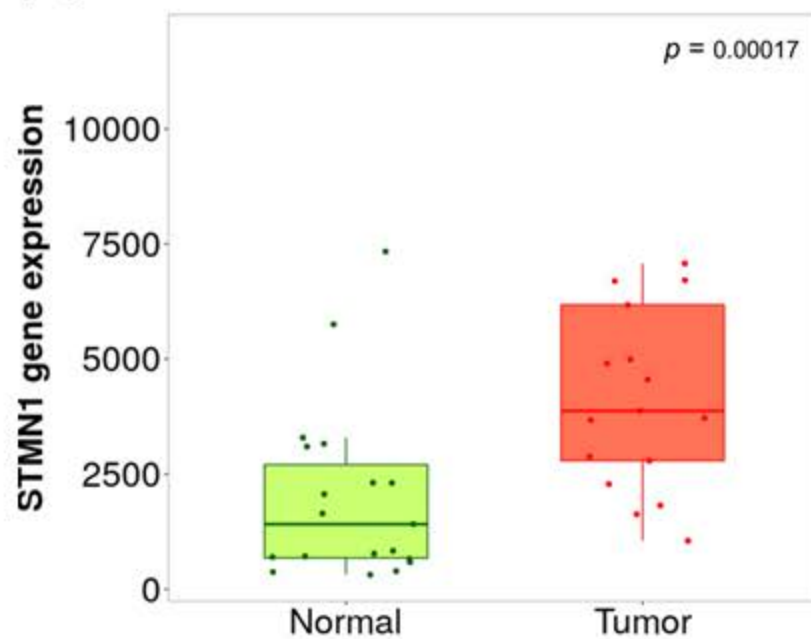

(F)

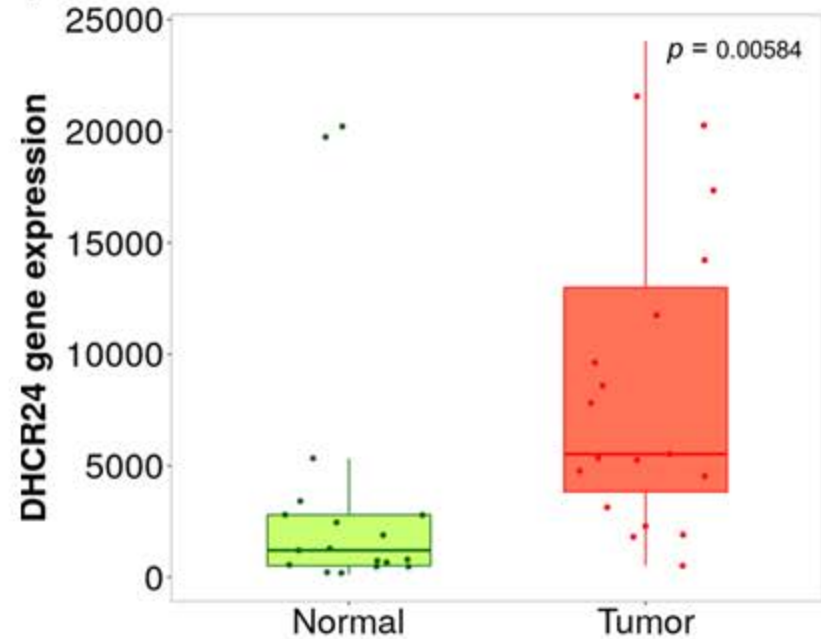

Supplement: Supplementary file 1 — Figure S1. [file CAM4-13-e70349-s002.pdf]
