# Supplementary material for: A Novel Gene Expression Scoring System Predicts Recurrence in Non‐Muscle‐Invasive Bladder Cancer Patients
Source: Cancer Med. 2024 Nov 14;13(22):e70349. doi: 10.1002/cam4.70349 (PMC11561421; doi:10.1002/cam4.70349)
Supplement: Supplementary file 3 — Data S1. [file CAM4-13-e70349-s001.docx]

**LIST OF SUPPLEMENTARY MATERIALS**

**Table S1. Microarray analysis and univariate Cox regression analysis of 6 genes for disease recurrence in 89 patients.**

**Figure S1. Expression levels of 6 genes in normal and bladder cancer tissues analyzed using TNMplot.com.** (A) CDCA3, (B) XRCC2, (C) ANLN, (D) MTHFD2, (E) STMN1, (F) DHCR24.
